# Integrated In Silico and In Vivo Evaluation of a Tetravalent SARS-CoV-2 RBD–Fc Fusion Vaccine with Broad Cross-Variant Antibody Responses

**DOI:** 10.3390/vaccines13121244

**Published:** 2025-12-15

**Authors:** Ahmad Bakur Mahmoud, Renad M. Alhamawi, Mustafa Yassin Taher, Awadh S. Alsubhi, Mekky M. Abouzied, Heba M. Zahid, Mohammed Abdullah Alotaibi, Nada Almarghalani, Khulood Alotaibi, Abdulrahman Habash, Shaker Ahmed Alsharif, Almohanad Alkayyal

**Affiliations:** 1 Department of Clinical Laboratory Sciences, College of Applied Medical Sciences, Taibah University, Madinah 42353, Saudi Arabia; rhamawi@taibahu.edu.sa (R.M.A.); mtaher@taibahu.edu.sa (M.Y.T.); assubhi@taibahu.edu.sa (A.S.A.); hzahid@taibahu.edu.sa (H.M.Z.); 2 Health and Life Research Center, Taibah University, Madinah 42353, Saudi Arabia; mabouzied@taibahu.edu.sa (M.M.A.); mhdalot@gmail.com (M.A.A.); nada.almarghalani@gmail.com (N.A.); kalotaibi0179@stu.kau.edu.sa (K.A.); 3 Department of Pharmacology and Toxicology, College of Pharmacy, Taibah University, Madinah 42353, Saudi Arabia; 4 King Abdullah International Medical Research Center, Jeddah 22384, Saudi Arabia; 5 College of Science, King Abdulaziz University, Jeddah 22384, Saudi Arabia; 6 Pharmaceutical Care Division, King Faisal Specialist Hospital & Research Centre, Madinah 42353, Saudi Arabia; ahabash@kfshrc.edu.sa; 7 Chest Department, King Fahad General Hospital, Madinah 42353, Saudi Arabia; shaalsharif@moh.gov.sa; 8 Department of Medical Laboratory Technology, Faculty of Applied Medical Sciences, University of Tabuk, Tabuk 71491, Saudi Arabia

**Keywords:** SARS-CoV-2, RBD–Fc fusion vaccine, protein subunit vaccine, cross-reactive antibodies, Omicron BA.4/BA.5, immune simulation, variant RBD binding, HEK293 expression

## Abstract

**Background/Objectives:** SARS-CoV-2 continues to generate antigenically divergent variants that reduce the breadth of existing vaccine-induced antibody responses. Fc-fusion subunit vaccines offer advantages in stability, antigen display, and Fc-mediated immune engagement. This study aimed to design and evaluate a tetravalent RBD–Fc fusion construct incorporating RBDs from Wuhan-Hu-1 and Omicron BA.4/BA.5 and to determine whether this configuration can induce broad antibody recognition across SARS-CoV-2 variants. The objective was to assess its feasibility, biochemical properties, and initial immunogenicity. **Methods:** Immune responses to the construct were first assessed using the C-ImmSim simulation platform. The full-length fusion was synthesized, subcloned into pcDNA3.1(+), expressed in HEK293 cells, and purified by Protein G affinity chromatography. Protein integrity was evaluated by reducing SDS–PAGE. BALB/c mice (female, 8 weeks) were immunized with a prime–boost–boost schedule, and sera were analyzed by ELISA, considering binding to Wuhan-Hu-1, Omicron BA.4/BA.5, and a panel of RBD variants. **Results:** In silico analysis predicted coordinated antigen clearance, class switching, memory B- and CD4^+^ T-cell formation, and transient cytokine induction. The recombinant protein was expressed efficiently, yielding a major ~56 kDa band and a ~23 kDa RBD fragment. Vaccinated mice generated strong IgG responses to Wuhan-Hu-1 and BA.4/BA.5 RBDs and showed broad binding to major variant RBDs. **Conclusions:** The tetravalent RBD–Fc fusion vaccine was successfully produced and elicited broad antibody binding across SARS-CoV-2 variants, supporting its potential as a versatile protein-based vaccine platform.

## 1. Introduction

SARS-CoV-2, the causative agent of COVID-19, is an enveloped, positive-sense RNA betacoronavirus. Viral entry depends on the trimeric spike (S) glycoprotein, which binds the angiotensin-converting enzyme 2 (ACE2) receptor on host cells [1]. The receptor-binding domain (RBD) within the S1 subunit mediates ACE2 engagement and is the main target of many neutralizing antibodies induced by infection or vaccination [2]. Due to its well-defined structure and functional importance, the RBD has become the primary antigen for the design of recombinant subunit vaccines [2].

First-generation COVID-19 vaccines based on the spike sequence of the Wuhan-Hu-1 strain have significantly reduced the risk of severe disease and death. However, SARS-CoV-2 has continued to evolve, giving rise to variants of concern such as Beta, Delta and Omicron [3]. Many of these variants carry multiple amino-acid substitutions in the RBD at positions including K417, E484, L452 and N501 [4]. These changes can reduce neutralization by sera from convalescent individuals and from vaccine recipients primed with ancestral spike-based vaccines. Omicron sublineages, including BA.4 and BA.5, show substantial antigenic divergence from Wuhan-Hu-1, highlighting the need for vaccine platforms that maintain activity across antigenically distinct RBDs rather than relying solely on a single strain [5].

Protein subunit vaccines, and RBD-based vaccines in particular, are one approach to this problem. Recombinant proteins can be manufactured using established processes, combined with licensed adjuvants and stored under standard refrigerated conditions [6]. Several RBD subunit vaccines and RBD-based constructs have shown favorable safety and immunogenicity profiles and protective efficacy in animal models and in humans [7]. A further improvement is the use of Fc-fusion formats, in which the viral RBD is genetically fused to a human IgG Fc fragment. In these designs, the Fc region forms a disulfide-linked dimer, so the fusion protein displays two copies of the RBD. The Fc portion retains interaction with the neonatal Fc receptor (FcRn) and Fcγ receptors, which can influence serum half-life and uptake by antigen-presenting cells [8]. Preclinical studies have shown that SARS-CoV-2 RBD–Fc vaccines elicit high levels of RBD-specific binding and neutralizing antibodies and protect animals against viral challenge [8].

Clinical data now support the feasibility of Fc-based COVID-19 vaccines in humans [9]. AKS-452 is a recombinant subunit vaccine consisting of an Fc fusion between the SARS-CoV-2 spike RBD and human IgG1 Fc, formulated with an oil-in-water adjuvant. Phase I/II and phase II booster studies reported that AKS-452 was generally well tolerated and induced robust RBD-specific IgG responses, including cross-reactive antibodies to several SARS-CoV-2 variants [9,10]. These results, together with supportive preclinical data, indicate that RBD–Fc constructs are a practical protein subunit vaccine platform for SARS-CoV-2.

Fc-fusion antigens have also been explored as vaccine candidates for other viral pathogens. For respiratory syncytial virus (RSV), influenza, and Fc-fusion constructs have also been applied to other coronaviruses: MERS-CoV RBD–Fc proteins produced in mammalian cells have shown strong immunogenicity and protective efficacy in animal models [11,12,13]. Taken together, these studies suggest that Fc-fusion designs can be adapted across diverse viral glycoproteins and can enhance the performance of protein subunit vaccines at the preclinical and early clinical stages.

Recent studies on multivalent and mosaic coronavirus vaccines have shown that co-displaying distinct RBDs on a single scaffold can broaden antibody recognition and strengthen coverage across antigenically divergent SARS-CoV-2 lineages [14,15,16]. Nanoparticle-based mosaic RBD vaccines and chimeric spike constructs developed over the past several years consistently report enhanced cross-variant binding and neutralization compared with monovalent immunogens [14,15,16,17,18], supporting the broader principle that simultaneous presentation of variant antigens can influence the breadth of the antibody response. Guided by these observations, our design incorporates the Wuhan-Hu-1 and Omicron BA.4/BA.5 RBDs within a single Fc-fusion immunogen, enabling concurrent presentation of both antigenic domains in a defined molecular configuration. The use of a single multivalent Fc-fusion protein also provides practical advantages for manufacturing, purification, and formulation, offering a streamlined approach compared with producing multiple independent RBD–Fc components.

## 2. Materials and Methods

### 2.1. In Silico Immune Simulation

Predicted immune responses to the tetravalent RBD–Fc vaccine were assessed using the C-ImmSim server (https://kraken.iac.rm.cnr.it/C-IMMSIM/index.php; accessed on 25 May 2025 and 3 November 2025). Simulations were run with the human host option, with HLA alleles set to HLA-A*02:01 and HLA-B*07:02 for MHC class I and HLA-DRB1*01:01 for MHC class II. The random seed was fixed at 12,345, the simulation volume was kept at the default 10 µL, and the total simulation length was 250 time steps. In C-ImmSim, a single time step corresponds to 8 h of real time; thus, the three injections were implemented at time steps 1, 63, and 105 to approximate a prime–boost–boost schedule on days 0, 21, and 35, and the simulation covered ~83 days in total. All remaining parameters, including baseline immune-cell numbers, mutation and proliferation rates, antigen-processing options, and cytokine settings, were left at the model default values. A detailed summary of all simulation inputs is provided in Supplementary Table S1. The full amino-acid sequence of the fusion construct (RBD_Fc_RBD_fullfusion) was used as the input antigen. The construct comprised an N-terminal signal peptide, the SARS-CoV-2 Wuhan-Hu-1 RBD (residues 319–541), a glycine–serine linker, the human IgG1 Fc region (hinge–CH2–CH3), a second glycine–serine linker, and the Omicron BA.4/BA.5 RBD (residues 319–541). Output parameters included antigen load, immunoglobulin isotypes, total and memory B cells, total and memory CD4^+^ T cells, plasma B cells, and cytokines (IL-2, IFN-γ, IL-4, IL-6, TNF-α, IL-10, TGF-β). No additional processing was applied prior to plotting.

### 2.2. Plasmid Construction

A tetravalent RBD–Fc expression cassette was designed to encode, in linear order, a human albumin signal peptide, the SARS-CoV-2 Wuhan-Hu-1 RBD (residues 319–541), a (GGGGS)\_3_ linker, the human IgG1 hinge–CH2–CH3 Fc region, a second (GGGGS)\_3_ linker, and the SARS-CoV-2 Omicron BA.4/BA.5 RBD (residues 319–541). The coding sequence was codon-optimized for human expression, synthesized, and subcloned into pcDNA3.1(+) under the CMV promoter by GenScript, Singapore, according to specifications provided by the authors.

### 2.3. Expression and Purification of Tetravalent RBD–Fc

HEK293 cells (InvivoGen, San Dieg, CA, USA) were cultured under standard conditions and transiently transfected with the pcDNA3.1(+)-RBD–Fc construct using Lipofectamine 2000 (Thermo Fisher Scientific, Waltham, MA, USA) following the manufacturer’s instructions. Plasmid DNA and Lipofectamine were mixed in serum-free medium, allowed to form complexes, and added to HEK293 cells at the recommended ratio. Culture supernatants were harvested five days post-transfection, clarified by centrifugation, and filtered through 0.22 μm membranes. The fusion protein was purified by Protein G affinity (Millipore Sigma, USA), eluted in low-pH buffer, immediately neutralized, and concentrated. Buffer exchange into PBS was performed using Amicon Ultra 100 kDa MWCO centrifugal filters (Millipore Sigma, St. Louis, MO, USA).

### 2.4. Protein Quantification, SDS–PAGE, and ACE2–Fc Western Blot

Protein concentration of purified tetravalent RBD–Fc was determined using the Bradford assay (Thermo Fisher Scientific, USA) with bovine serum albumin as the standard. Purified material was analyzed under reducing conditions by SDS–PAGE using Coomassie blue staining. To confirm the presence of RBD, reduced samples were subjected to Western blotting and probed with recombinant human ACE2 (mouse IgG1 Fc tag; ACROBiosystems, Way Newark, DE, USA), followed by HRP-conjugated anti-mouse IgG1 for detection. A titration of vaccine protein (0.50, 0.25, and 0.12 µg) was processed in parallel.

### 2.5. Animals and Immunization

Eight-week-old female BALB/c mice were used for immunogenicity testing. Animals were randomly assigned to vaccinated and control groups, and the number of mice in each group is shown as ‘n’ in the figure legends). All procedures were conducted in accordance with institutional policies for the care and use of laboratory animals and were approved by the Research Ethics Committee, College of Pharmacy, Taibah University (Approval No. COPTU-REC-170-20250823). Vaccinated mice received 50 μg of the tetravalent RBD–Fc protein formulated 1:1 (*v*/*v*) with AddaVax (InvivoGen, USA) on days 0, 21, and 35. Control mice received AddaVax alone on the same schedule. Sera were collected on day 60 and stored at ≤−20 °C until analysis

### 2.6. ELISA for RBD Binding and Cross-Reactivity

High-binding 96-well plates were coated overnight at 4 °C with recombinant RBDs diluted in PBS. Plates were blocked with PBS containing 3% BSA, and mouse sera were serially diluted as indicated in the figure legends. Bound IgG was detected using HRP-conjugated anti-mouse IgG and TMB substrate (Thermo Fisher Scientific, USA), and absorbance was measured at 450 nm. Background subtraction was performed using PBS-coated control wells from the same plate. Recombinant proteins (Sino Biological, Beijing, China) included SARS-CoV-2 RBDs from Wuhan-Hu-1 (WT), K417N, L452R/E484Q, E484K, N501Y, B.1.1.529 (Omicron, Tokyo, Japan), and BA.4/BA.5/BA.5.2. All antigens were tested under identical assay conditions.

### 2.7. Statistical Analysis

Data were analyzed in GraphPad Prism 10 (v10.6.1) and are reported as mean ± s.d., with sample sizes indicated in the legends. Group comparisons were performed using two-way ANOVA with Šídák’s correction for multiple testing. A *p*-value < 0.05 was considered statistically significant.

## 3. Results

### 3.1. In Silico Prediction of Immune Responses Induced by the Tetravalent RBD–Fc Vaccine

Before moving to experimental validation, the immunogenicity of the designed tetravalent RBD–Fc fusion vaccine was first assessed in silico using the C-ImmSim immune simulation platform. The construct encoded the receptor-binding domains (RBDs) of SARS-CoV-2 Wuhan-Hu-1 and Omicron BA.4/5, fused to a human IgG1 Fc fragment through flexible linkers. In C-ImmSim, each time step represents approximately 8 h of simulated immune activity [19,20]. Injection times were therefore placed at steps 1, 63, and 105 to approximate the 0-, 21-, and 35-day spacing that were used in mice later on. The total simulation length was kept at 250 time steps, the standard duration recommended for observing full primary and secondary immune dynamics in this model. The goal was to predict antigen clearance, antibody kinetics, and cellular immune responses, including T-helper, B-cell, and cytokine activation profiles over an 80-day simulation period. The simulation was used as an exploratory tool to examine expected qualitative patterns of antigen clearance and adaptive immune activation for this specific construct prior to in vivo testing, rather than as a predictive model of quantitative immune kinetics.

**Figure 1:**
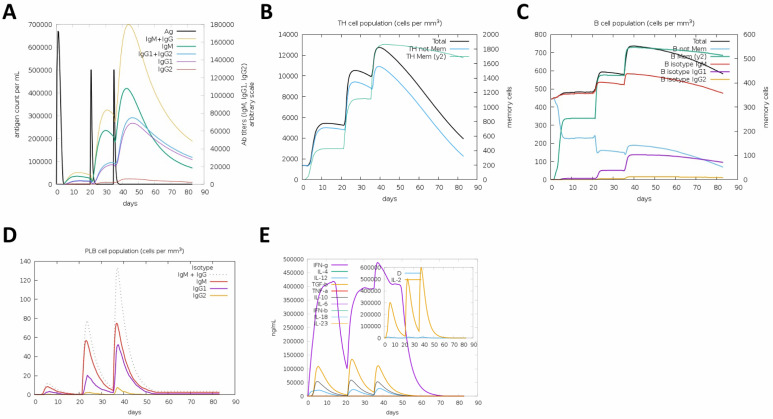
**In silico simulation of immune responses induced by the tetravalent RBD–Fc vaccine.** (**A**) Simulated antigen kinetics following a prime–boost–boost schedule. Antigen levels peak after each exposure and decline as antibodies rise. (**B**) CD4^+^ T-helper cell dynamics showing expansion after each dose and formation of memory T cells. (**C**) Total and memory B-cell responses, with marked increases following booster immunizations. (**D**) Plasma B-cell kinetics demonstrating peaks after the first and second boosts. (**E**) Cytokine profiles showing transient induction of IL-2 and IFN-γ, with moderate IL-4, IL-6, TNF-α, and later regulatory cytokines (IL-10, TGF-β). Together, the simulation predicts a coordinated adaptive immune response to the tetravalent RBD–Fc construct.

The simulation results demonstrated a well-coordinated and robust adaptive immune response following vaccination (Figure 1A–E). In Figure 1A, antigen concentration (Ag) sharply increased after each injection and was followed by rapid clearance, accompanied by sequential antibody production. IgM rose transiently after the first dose, whereas IgG1 and IgG2 titers progressively increased after each booster, reflecting successful class switching and memory response development. Figure 1B shows the dynamics of CD4^+^ T-helper cells, which expanded markedly after each dose, with a substantial proportion transitioning into long-lived memory cells. This increase coincided with the strong secondary antibody responses, indicating effective T–B cell cooperation. In Figure 1C, total and memory B lymphocytes rose after the primary and booster doses, with the IgG1 and IgG2 isotypes dominating over IgM, confirming affinity maturation and isotype switching. Figure 1D presents the plasma B-cell population, which peaked following the first and second boosts and contributed to the rise in IgG1 and IgG2 antibody titers. Finally, Figure 1E illustrates the cytokine profiles, highlighting transient spikes in IL-2 and IFN-γ, accompanied by moderate levels of IL-4, IL-6, and TNF-α. These cytokines are consistent with Th1/Th2-balanced immune activation. The presence of regulatory cytokines such as IL-10 and TGF-β at later stages indicates an appropriate resolution of the immune response.

Together, these findings predict that the tetravalent RBD–Fc fusion vaccine elicits a strong and balanced immune response characterized by antigen clearance. The simulation supports the construct’s potential as an immunogenic and safe subunit vaccine, providing the rationale for subsequent experimental production and validation.

### 3.2. Construction, Expression, and Purification of the Recombinant Tetravalent RBD–Fc Fusion Vaccine

To experimentally produce the designed tetravalent RBD–Fc fusion vaccine, we constructed an expression cassette encoding the RBDs from both the ancestral SARS-CoV-2 strain (Wuhan-Hu-1) and the Omicron BA.4/5 variant. The two RBDs were joined by flexible (GGGGS)_3_ linkers and fused through a human IgG1 Fc fragment that included the hinge, CH2, and CH3 regions. An albumin signal peptide was placed at the N-terminus to direct efficient secretion in mammalian cells (HEK293). The DNA construct was synthesized and subcloned into pcDNA3.1(+) and transiently expressed in HEK293 cells.

**Figure 2:**
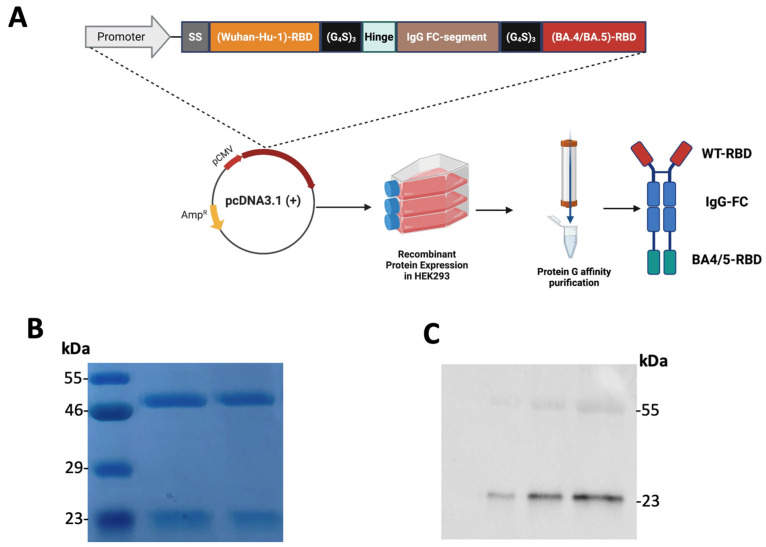
**Construction, expression, and purification of the tetravalent RBD–Fc fusion vaccine.**(**A**) Schematic representation of the vaccine design and purification workflow, showing the N-terminal signal peptide, Wuhan-Hu-1 RBD, flexible linker, human IgG1 Fc region, a second linker, and Omicron BA.4/5 RBD, followed by expression in HEK293 cells and Protein G affinity purification. (**B**) Reducing SDS–PAGE with Coomassie blue staining showing a ~56 kDa band and a smaller ~23 kDa band. (**C**) ACE2 reactivity of the purified protein assessed by Western blot. Vaccine samples (0.50, 0.25, and 0.12 µg) were probed with recombinant human ACE2 fused to a mouse IgG1 Fc tag, followed by HRP-conjugated anti-mouse IgG.

Five days post-transfection, the supernatant from transfected HEK293 cells was harvested, centrifuged, and filtered through a 0.22 µm membrane filter. The recombinant fusion protein was purified by affinity chromatography using Protein G (Figure 2A). to confirm the integrity and purity of the expressed protein, purified protein samples were separtaed using reducing SDS–PAGE followed by Coomassie blue staining. (Figure 2B) shows two distinct bands a dominant band at approximately 56 kDa as expected, and a smaller band at approximately 23 kDa, consistent with the size of RBD fragment. To confirm the presence of RBD in the lower-molecular-weight species, we performed a Western blot using recombinant human ACE2 bearing a mouse IgG1 Fc tag as the probe. ACE2 produced a clear signal at ~23 kDa across a titration of vaccine protein (0.50, 0.25, and 0.12 µg), indicating that this band contains RBD fragment (Figure 2C). A faint ACE2-reactive signal was also detected between ~46–55 kDa, which may reflect a partially cleaved intermediate that retains some RBD structure; a minor contribution from cross-recognition of the human Fc region by the anti-mouse secondary antibody cannot be excluded.

These results confirm the successful molecular assembly, expression, secretion, and purification of the tetravalent RBD–Fc fusion vaccine in a mammalian system. The integrity of both the Fc and RBD regions supports its suitability for downstream immunogenicity evaluation in in vivo and in silico studies.

### 3.3. In Vivo Evaluation of Serum Antibody Responses Following Tetravalent RBD–Fc Vaccination

**Figure 3:**
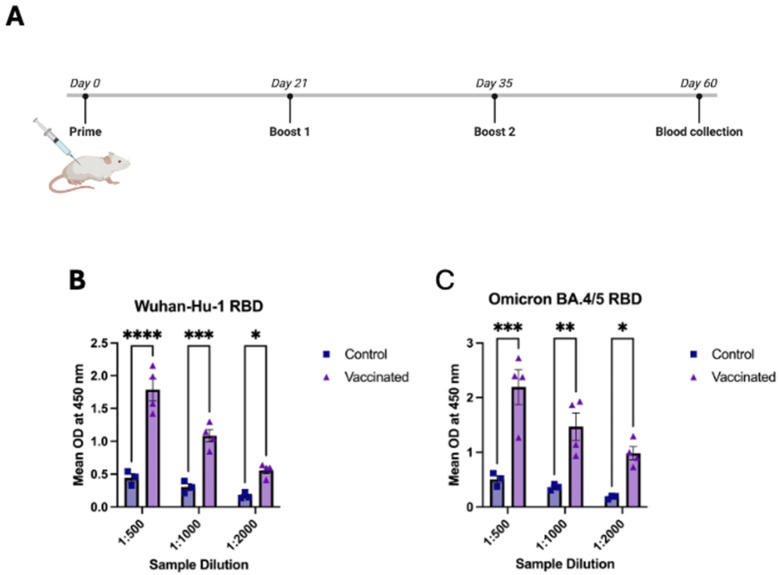
**Serum antibody responses to Wuhan-Hu-1 and Omicron BA.4/5 RBDs after vaccination** (**A**) **Vaccination schedule showing prime immunization on day 0 followed by booster doses on days 21 and 35, with serum collection on day 60.** (**B**) ELISA binding of sera from vaccinated (n = 4) and control (n = 3) mice to Wuhan-Hu-1 RBD across serial dilutions (1:500–1:2000). Vaccinated mice showed significantly higher IgG binding at all measured dilutions. (**C**) ELISA binding of the same sera to Omicron BA.4/5 RBD using the same dilution series. Vaccinated mice displayed consistently higher binding than controls. Data are presented as mean ± s.d. Asterisks indicate statistical significance: *p* < 0.05 (*), *p* < 0.01 (**), *p* < 0.001 (***), and *p* < 0.0001 (****).

To determine whether the recombinant tetravalent RBD–Fc vaccine could elicit an antigen-specific humoral response, BALB/c mice were immunized following a prime–boost–boost schedule on days 0, 21, and 35, with serum collected on day 60 (Figure 3A). Enzyme-linked immunosorbent assays (ELISA) were performed using recombinant Wuhan-Hu-1 RBD (RBD-WT) and Omicron BA.4/5 RBD antigens to measure the binding activity of serum antibodies at serial dilutions.

Vaccinated mice developed strong binding antibody responses against both RBD-WT and RBD-BA.4/5 antigens (Figure 3B,C). For RBD-WT, vaccinated serum displayed significantly high binding capacity to Wuhan-Hu-1 RBD compared with controls at dilutions of 1:500 (**** *p* < 0.0001), 1:1000 (*** *p* < 0.001), and 1:2000 (* *p* < 0.05). A similar pattern was observed with RBD-BA.4/5, where binding was markedly elevated in the vaccinated group at 1:500 (*** *p* < 0.001), 1:1000 (** *p* < 0.01), and 1:2000 (* *p* < 0.05). As all plates were coated with isolated SARS-CoV-2 RBD antigens, the signals measured primarily reflect RBD-specific binding antibodies. Any responses directed against the Fc scaffold or linker regions would not be captured in this assay format.

These findings demonstrate that the tetravalent RBD–Fc vaccine effectively induces robust antibody responses capable of recognizing both Wuhan-Hu-1 and Omicron (BA.4/5) RBD antigens.

### 3.4. Cross-Reactive Binding of Vaccinated Mouse Sera to SARS-CoV-2 RBD Variants

We next evaluated the antigenic breadth of the tetravalent RBD–Fc vaccine by testing sera from vaccinated mice against a representative panel of SARS-CoV-2 receptor-binding domains (RBDs) from variants of concern. The set included the Wuhan-Hu-1 (WT), Alpha/Beta/Gamma (N501Y), Beta/Gamma (E484K), Beta (K417N), Kappa (L452R/E484Q), and Omicron sublineages BA.4/BA.5 and B.1.1.529. This study examined whether the antibodies generated by the tetravalent vaccine could recognize both the original and variants of concern.

**Figure 4:**
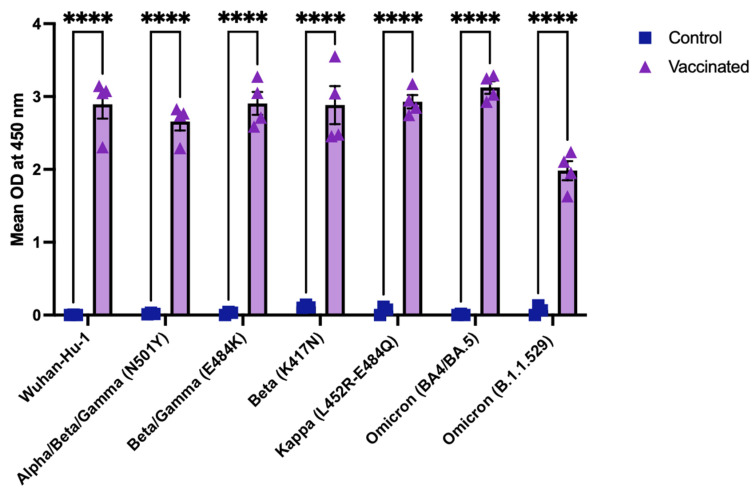
**Cross-reactive binding of vaccinated mouse sera to SARS-CoV-2 RBD variants.** ELISA binding of sera from vaccinated (n = 4) and control (n = 3) mice to a panel of recombinant RBDs: Wuhan-Hu-1 (WT), N501Y, E484K, K417N, L452R/E484Q, Omicron BA.4/BA.5, Omicron B.1.1.529. Data are shown as mean ± s.d. Asterisks indicate statistical significance: *p* < 0.0001 (****).

Sera collected from vaccinated mice displayed strong and broad binding across all tested SARS-CoV-2 RBD variants (Figure 4). Binding to the Wuhan-Hu-1 RBD was comparable to that observed for the major variants carrying substitutions in key neutralizing epitopes such as N501Y, E484K, and L452R/E484Q. Despite the structural divergence introduced by multiple mutations in Omicron BA.4/BA.5 and B.1.1.529, serum reactivity remained strong, demonstrating that antibodies elicited by the tetravalent vaccine target conserved regions within the RBD.

Together, these results demonstrate that the tetravalent RBD–Fc vaccine induces broad-spectrum antibody responses that extend beyond SARS-CoV-2 variant-specific recognition.

## 4. Discussion

The aim of this study was to examine whether two distinct SARS-CoV-2 RBDs, Wuhan-Hu-1 and Omicron BA.4/BA.5, could be combined in a single Fc-fusion protein and still produce a broad antibody response. The construct was produced in HEK293 cells and yielded a protein that retained RBD antigenicity and triggered a clear humoral response in mice. The in vivo immunization data show that this format elicits strong binding antibody responses to both RBDs, and that vaccinated sera recognize a broad panel of SARS-CoV-2 variant RBDs. These observations indicate that the linked RBD–Fc arrangement remains immunogenic and that the two RBD components can be presented together without apparent interference.

Work in the last few years has shown that multivalent and mosaic coronavirus vaccines can broaden the antibody repertoire by presenting antigenically distinct RBDs in one immunogen. Examples include RBD nanoparticles displaying mixtures of sarbecovirus RBDs and engineered chimeric spike proteins, both of which have produced wider recognition of variant RBDs in preclinical models. Our approach relies on the same general idea but uses a soluble Fc-fusion format instead of a nanoparticle scaffold. Fc-fusion proteins are well established in clinical manufacturing, and this provides a practical advantage: a single molecule carrying two RBDs can be produced and purified using standard antibody workflows, which avoids generating and formulating multiple separate RBD components. Because Fc-fusion proteins are well established in therapeutic antibody manufacturing, this approach offers a practical route for production, purification, and formulation using existing antibody development pipelines.

The immunogenicity profile observed in mice suggests that simultaneous presentation of Wuhan-Hu-1 and Omicron BA.4/BA.5 RBDs within one Fc dimer can support recognition of both antigenic lineages. The sera also bound RBDs carrying substitutions such as N501Y, E484K, L452R/E484Q, and K417N, all of which have been associated with immune evasion. Binding alone does not determine neutralization, but the range of reactivity gives a useful indication that the construct exposes epitopes shared across different variant lineages. These findings are encouraging for the continued exploration of multivalent Fc-based vaccine formats. Because ELISA measurements in this study were performed using isolated RBD antigens, the antibody responses observed predominantly reflect recognition of the viral RBD regions. Fc- or linker-specific antibodies were not evaluated, and C-ImmSim likewise models the full fusion protein without resolving responses to individual domains. Prior studies of Fc-fusion vaccines for influenza, RSV, and SARS-CoV-2 have reported that immune responses are largely directed toward the viral antigen component rather than the Fc region, supporting the feasibility of this format [8,9,10,11,12]. Future iterations of this platform will examine Fc-specific responses and linker variants more directly.

The study has planned limits. The experiments were set up to obtain an initial view of immunogenicity rather than a full functional assessment. Neutralization assays, T-cell analyses, and challenge studies were not included here and will be essential for understanding the actual protective potential of this design. The work also did not compare the tetravalent construct directly with monovalent or mixed RBD–Fc formats. Such comparisons will be needed to determine whether physical linkage offers any immunological benefit over co-administration of single RBD-Fc proteins.

In summary, the results show that a multivalent Fc-fusion protein carrying two divergent SARS-CoV-2 RBDs can be stably expressed and can stimulate antibody responses that recognize a wide range of variant RBDs. The approach is straightforward to produce and aligns well with established Fc-based manufacturing platforms. The data support continued development of this format with a focus on functional neutralization, comparative immunization studies, and evaluation in broader preclinical models.

## 5. Conclusions

This study describes the design and preclinical assessment of a tetravalent RBD–Fc fusion vaccine incorporating RBDs from Wuhan-Hu-1 and Omicron BA.4/BA.5 within a single recombinant construct. The integrated in silico, in vitro, and in vivo analyses consistently showed that the vaccine is stable, readily produced in mammalian cells, and capable of inducing strong serum IgG responses. The antibody binding profile demonstrated recognition of both ancestral and Omicron RBDs, along with broad cross-reactivity to multiple SARS-CoV-2 variants. These findings support the feasibility of using multi-RBD Fc-fusion formats to enhance the breadth of antibody responses. Further work will be required to characterize neutralizing activity, cellular immunity, and protective efficacy in challenge models, but the current results provide a solid foundation for advancing this platform in future studies.

## Data Availability

The original contributions presented in the study are included in the article, further inquiries can be directed to the corresponding authors.

## Supplementary Materials

The following supporting information can be downloaded at: https://www.mdpi.com/article/10.3390/vaccines13121244/s1, Table S1: C-ImmSim simulation settings used in this study.

## Abbreviations

The following abbreviations are used in this manuscript:

**Abbreviation**	**Definition**
RBD	Receptor-binding domain
Fc	Fragment crystallizable region
SDS–PAGE	Sodium dodecyl sulfate–polyacrylamide gel electrophoresis
PBS	Phosphate-buffered saline
BSA	Bovine serum albumin
ELISA	Enzyme-linked immunosorbent assay
HRP	Horseradish peroxidase
TMB	3,3′,5,5′-Tetramethylbenzidine
WT	Wild type
MWCO	Molecular weight cut-off
IL	Interleukin
IFN-γ	Interferon gamma
TNF-α	Tumor necrosis factor alpha
TGF-β	Transforming growth factor beta
CMV	Cytomegalovirus promoter
OD_450_	Optical density at 450 nm
HEK293	Human embryonic kidney 293 cells
C-ImmSim	Computational Immune Simulation Platform
