# Supplementary material for: Integrated In Silico and In Vivo Evaluation of a Tetravalent SARS-CoV-2 RBD–Fc Fusion Vaccine with Broad Cross-Variant Antibody Responses
Source: Vaccines (Basel). 2025 Dec 15;13(12):1244. doi: 10.3390/vaccines13121244 (PMC12737504; doi:10.3390/vaccines13121244)
Supplement: Supplementary file 1 [file vaccines-13-01244-s001.zip › vaccines-4017403-Supplementary.pdf]

Supplementary Table S1. C-ImmSim simulation settings used in this study.

Parameter	Value
Server URL	https://kraken.iac.rm.cnr.it/C-IMMSIM/index.php
Access date	25 May 2025 and 3 November 2025
Host organism	Human
HLA alleles (MHC I)	HLA-A*02:01, HLA-B*07:02
HLA alleles (MHC II)	HLA-DRB1*01:01
Antigen sequence	Full amino-acid sequence of the tetravalent RBD–Fc fusion construct
Random seed	12345
Simulation volume	10 µL (default)
Total time steps	250
Injection time steps	1, 63, 105
Antigen dose	Constant across injections
Mutation/proliferation rates	Default
Cell population baselines	Default
Cytokine settings	Default
